# Characterization of Donor Variability for γδ T Cell *ex vivo* Expansion and Development of an Allogeneic γδ T Cell Immunotherapy

**DOI:** 10.3389/fmed.2020.588453

**Published:** 2020-11-13

**Authors:** Rebecca E. Burnham, Jaquelyn T. Zoine, Jamie Y. Story, Swetha N. Garimalla, Greg Gibson, Aaron Rae, Erich Williams, Lisa Bixby, David Archer, Christopher B. Doering, H. Trent Spencer

**Affiliations:** ^1^Department of Pediatrics, Aflac Cancer and Blood Disorders Center, Emory University School of Medicine, Atlanta, GA, United States; ^2^Molecular and Systems Pharmacology Program, Graduate Division of Biological and Biomedical Sciences, Emory University School of Medicine, Atlanta, GA, United States; ^3^Cancer Biology Program, Graduate Division of Biological and Biomedical Sciences, Emory University School of Medicine, Atlanta, GA, United States; ^4^School of Biological Sciences, Georgia Institute of Technology, Atlanta, GA, United States

**Keywords:** gamma delta (γδ) T cells, allogeneic cell products, *ex vivo* expanded T cells, serum free expansion, mixed cell product

## Abstract

Gamma delta (γδ) T cells recently emerged as an attractive candidate for cancer immunotherapy treatments due to their inherent cytotoxicity against both hematological and solid tumors. Moreover, γδ T cells provide a platform for the development of allogeneic cell therapies, as they can recognize antigens independent of MHC recognition and without the requirement for a chimeric antigen receptor. However, γδ T cell adoptive cell therapy depends on *ex vivo* expansion to manufacture sufficient cell product numbers, which remains challenging and limited by inter-donor variability. In the current study, we characterize the differences in expansion of γδ T cells from various donors that expand (EX) and donors that fail to expand, i.e., non-expanders (NE). Further, we demonstrate that IL-21 can be used to increase the expansion potential of NE. In order to reduce the risk of graft vs. host disease (GVHD) induced by an allogeneic T cell product, αβ T cell depletions must be considered due to the potential for HLA mismatch. Typically, αβ T cell depletions are performed at the end of expansion, prior to infusion. We show that γδ T cell cultures can be successfully αβ depleted on day 6 of expansion, providing a better environment for the γδ T cells to expand, and that the αβ T cell population remains below clinically acceptable standards for T cell-depleted allogeneic stem cell products. Finally, we assess the potential for a mixed donor γδ T cell therapy and characterize the effects of cryopreservation on γδ T cells. Collectively, these studies support the development of an improved allogeneic γδ T cell product and suggest the possibility of using mixed donor γδ T cell immunotherapies.

## Introduction

Gamma delta (γδ) T cells are a unique and promising candidate for the development of cancer immunotherapy treatments. γδ T cells are key players in the innate immune system, inducing cytotoxicity directly through the expression of cell surface receptors such as the γδ T-cell receptor and natural killer group 2D (NKG2D) (1, 2). Upon activation, γδ T cells release cytotoxic cytokines and chemokines that directly contribute to the cytolysis of tumor cells (3, 4). Additionally, γδ T cells serve as modulators in the adaptive immune system and can target tumor cells indirectly by priming αβ T cells, recruiting B cells to assist with antibody production, and activating dendritic cells (5–7). A variety of cytotoxic mechanisms have been shown to contribute to γδ T cell anti-tumor activity in preclinical studies (8–12), and moderate patient responses to γδ T-cell immunotherapies have been observed in early phase clinical trials (13–20).

There are two main subsets of γδ T cells currently under investigation: Vδ1 and Vδ2. Vδ1 T cells, while enriched in tissues such as the intestine, colon, and dermis, comprise only a small percentage of circulating peripheral blood γδ T cells. While studies have shown that Vδ1 T cells may have increased cytotoxic potential against certain hematopoietic malignancies and solid tumors (21–23), challenges remain in developing a good manufacturing practice (GMP) compliant expansion protocol. Unlike Vδ2 T cells, Vδ1 T cells neither respond to amino-bisphosphonate (N-BP) nor phosphoantigen (pAG)-mediated stimulation (24–26). Protocols have been developed to successfully expand Vδ1 T cells using plant mitogens, cytokines and irradiated feeder cells (27–29), and some are advancing to clinical trials. In addition, recent evidence has implicated IL-17 producing Vδ1 T cells in promoting tumor progression (30, 31), revealing the importance of characterizing IL-17 production and ensuring that γδ T-cell therapies do not promote tumor growth. Given these challenges and the fact that clinical-grade N-BP and pAG are available and approved for use in humans, Vδ2 T cells have been a primary focus for clinical development.

Clinical trials using γδ T-cell based immunotherapies have tried to expand Vδ2 T cells *in vivo* through direct administration of stimulating agents (14–17) or *ex vivo* through the collection and stimulation of γδ T cells in a pool of peripheral blood mononuclear cells (PBMCs) (18–20). *In vivo* expansions of γδ T cells using a combination of the N-BP, zoledronate, and the cytokine, IL-2, have proved difficult due to the off-target expansion of regulatory T cells (32, 33) and dose-limiting toxicities associated with cytokine therapies (34). Due to these challenges, γδ T cells have been investigated in the context of adoptive cell transfer, in which autologous cells are expanded *ex vivo* and reinfused into the patient (13, 18, 19, 35, 36). This approach allows for the selective expansion of γδ T cells and the complete characterization of effector cells. However, the feasibility of adoptive cell transfer can be reduced due to challenges faced in expanding γδ T cells from patient derived PBMCs. Due to their ability to recognize target cells independently of human leukocyte antigen (HLA) mediated antigen presentation, γδ T cells are a viable candidate for allogeneic cell therapies in which third party donor cells are expanded *ex vivo* and either immediately infused into a patient or cryopreserved until needed for treatment. Expanding a clinically relevant number of γδ T cells from PBMCs remains a significant challenge in the development of an allogenic γδ T cell immunotherapy. Our lab has developed a GMP compliant protocol for the serum-free expansion of Vδ2 T cells from PBMCs using zoledronate and IL-2 (37, 38). However, we and others have reported significant donor to donor variability in the *ex vivo* expansion of γδ T cells (39, 40). Recent studies have shown up to an 80-fold difference between donor expansions, and while γδ T cells from some donors' PBMCs expand to comprise >90% of the total PBMC culture at the end of expansion, other donors never achieve more than 30% of the total culture (39). Understanding the differences in inter-donor variability in order to select donors whose PBMCs will successfully expand is essential for clinical use of γδ T cells in an allogeneic cell therapy setting.

The goals of these studies were to characterize the variability in the expansion of healthy donor γδ T cells and to optimize the expansion process for the development of a mixed donor γδ T cell immunotherapy. We report herein (i) a characterization of the variability in expansion of healthy donor γδ T cells; (ii) evidence that γδ T cell expansion from NE can be “rescued” with the addition of IL-21; (iii) optimization of the protocol for the depletion of αβ T cells during the expansion of γδ T cells, reducing the amount of reagents necessary for this procedure; (iv) the development of a novel allogeneic mixed γδ T cell immunotherapy; and (v) a characterization of the effects of cryopreservation on γδ T cells.

## Methods

### Expansion of γδ T Cells and Classification of Donors

Peripheral blood (40 mL) from 16 healthy donors was collected through the Emory Children's Clinical and Translational Discovery Core (IRB00101797). Donors were pre-selected based on self-reported levels of exercise and age. The 8 donors in the exercise category self-reported intense physical exercise between 4 and 7 days a week, while the 8 sedentary donors reported exercising 0–1 day a week. To reduce the impact of age on γδ T cell expansion, all donors that participated in this study were under the age of 35. PBMCs were isolated from whole blood via density gradient centrifugation with Ficoll-Paque Plus (GE Healthcare Life Sciences). After isolation, PBMCs were cultured in OpTmizer media (Life Technologies), supplemented with OpTmizer T-cell expansion supplement, 1% penicillin/streptomycin and 2 mM L-glutamine, and stimulated with zoledronate (Sigma-Aldrich) and IL-2 (Peprotech). Cell counts were performed using a Cellometer (Nexelcom) and cells were resuspended in fresh media at 1.5 × 10^6^ cells/mL every 3 days. Zoledronate (5 μM) and IL-2 (500 IU/mL) were added on day 0 and 3 of culture. IL-2 (1,000 IU/mL) was added on days 6, 9, and 12 of expansion. Flow cytometry was used to determine the percentage of γδ T cells in culture on days 0, 6, and 12 or 14 of expansion. Cell growth experiments were performed by plating 5 × 10^6^ cells for each donor and taking cell counts of the expansion every 3 days.

While testing the supplementation of other cytokines, all expansions received zoledronate (5 μM) and IL-2 (500 IU/mL) on day 0 and 3 of culture. The “normal” expansion conditions received 1,000 IU/mL of IL-2 on day 6, 9, and 12 of expansion. The other conditions received 10 ng/mL of IL-15 (Peprotech), IL-21 (Peprotech), or IL-15+IL-21 from day 0 through day 12 of expansion and 500 IU/mL of IL-2 on day 6, 9, and 12 of expansion. As stated above, cell growth experiments were performed by plating 5 × 10^6^ cells for each donor and taking cell counts of the expansion every 3 days.

### RNA-Sequencing

RNA-sequencing was performed on *ex vivo* expanded and cell sorted γδ T cells from 3 donors. Reads were aligned with Kallisto and transcripts per gene were collapsed to a gene. Kallisto reported 26,898 genes present and these genes were sorted to include genes present in 3 samples with an average of at least 0.2 transcripts per million reads (TPM), generating a list of 13,693 genes. A value of 1 was added to each collapsed TPM and the values were converted to log_2_ to create a relative expression range of 0 to 14.0.

### Flow Cytometry

Cells were washed with phosphate buffer saline (PBS) and spun at 300 × g in flow cytometry tubes. The supernatant was decanted and replaced with eBioscience Fixable Viability Dye eFluor780 (ThermoFisher) for 30 min. Cells were washed in 10 × PBS and resuspended with the appropriate antibodies. Antibodies from BD Biosciences include: BV421 Mouse Anti-Human CD3 (Clone UCHT1), PE Mouse Anti-Human γδ TCR (Clone 11F2), BUV395 Mouse Anti-Human CD56 (Clone NCAM16.2), BUV395 Mouse Anti-Human CD56, BV711 Mouse Anti-Human CD178 (Clone NOK-1), BV786 Mouse Anti-Human CD107a (Clone H4A3), BV480 Mouse Anti-Human CD3 (Clone UCHT1), APC-R700 Mouse Anti-Human CD56 (Clone NCAM16.2), BV711 Mouse Anti-Human CD27 (Clone M-T271), BUV496 Mouse Anti-Human CD16 (Clone 3G8), BV421 Mouse Anti-Human CD57 (Clone NK-1), BV786 Mouse Anti-Human PD1 (Clone EH12.1), PE-CF594 Mouse Anti-Human PDL1 (Clone MIH1), BUV737 Mouse Anti-Human FAS (Clone DX2), PE Mouse Anti-Human FASL (Clone NOK-1), and BUV395 Mouse Anti-Human CD107a (Clone H4A3). Cells were analyzed using a LSRII (BD Biosciences), an Aurora (CYTEK), and a BD FACSymphony (BD Biosciences).

### αβ T Cell Depletions

αβ T cell depletions were performed according to the manufacturer's protocol (Miltenyi Biotec). Briefly, cells were washed in autoMACS Rinsing Solution containing 0.5% BSA (Miltenyi Biotec) and spun at 300 × g for 5 min. Cells were incubated with Anti-TCRα/β-Biotin (Miltenyi Biotec) for 10 min at 4°C, then washed in autoMACS Rinsing Solution and filtered through a 0.4 μM filter. Cells were then incubated with Anti-Biotin Microbeads (Miltenyi Biotec) for 15 min at 4°C, washed in autoMACS Rinsing Solution, and passed through an LD Column (Miltenyi Biotec). After depletion, cells were counted and resuspended in OpTmizer Media with 1,000 IU/mL of IL-2. Flow cytometry was used to assess the efficiency of depletion as described above.

### Cytotoxicity Assays

Flow cytometry-based cytotoxicity assays were performed to test the *in vitro* cytotoxic potential of *ex vivo* expanded γδ T cells against a malignant cell line (26, 27). The target cell line used in this study was the chronic myelogenous leukemia cell line K562 (ATCC). Target cells were labeled with Violet Proliferation Dye 450 (BD Biosciences) and incubated with γδ T cells at effector to target cell ratios of 1:1 and 5:1 for 4 h at 37°C. Flow cytometry was used to measure target cell death, using the dead stain eBioscience Fixable Viability Dye eFluor 780 (ThermoFisher) and the early apoptosis stain Annexin V (BioLegend). In studies using the mixed γδ T cell product after thawing, additional target cell lines were tested including: Nomo-1, MOLT-4, SEM, Nalm-6, and Jurkats. The Nomo-1 and MOLT-4 cell lines were a gift from the laboratory of Dr. Douglas Graham (Emory University). The SEM and Nalm-6 cells lines were a gift from the laboratory of Dr. Curtis Henry (Emory University). The Jurkats cells were obtained from ATCC. γδ T cell cytotoxicity was calculated by subtracting the background cell death of each target cell line from each experimental sample.

### Cell Mixing

γδ T cells from individual donors were grown in culture through day 6 of expansion. After performing an αβ depletion on day 6, 2 × 10^6^ γδ T cells from 3 donors were mixed together at a ratio of 1:1:1 and expanded under normal conditions. Flow cytometry was used to assess the percentage of γδ T cells in culture to determine if γδ T cells from different donors could grow in culture together.

### Cryopreservation of γδ T Cells

To freeze, γδ T cells were washed with PBS and spun at 300 × g for 5 min. Cells were resuspended at a concentration of 1 × 10^7^ cells/mL in Human Albumin U.S.P. Albutein 5% (Grifols Therapeutics Inc.) and 10% DMSO. Cells were frozen at a rate of −1°C per minute and moved to liquid nitrogen storage when they reached a temperature of −80°C. To thaw, γδ T cells were removed from liquid nitrogen and incubated in a 37°C water bath. When the cells were nearly thawed, they were removed from the water bath and diluted in media. Cells were spun at 300 × g for 5 min and then resuspended in media containing IL-2 (1,000 IU/mL).

### Statistical Analysis

All figures and statistics were generated in GraphPad Prism Software, Version 8.2.1. Data were analyzed using a Student's *t-test* or a two-way analysis of variance (ANOVA) with Sidak's multiple comparisons *post hoc* tests. Corresponding tests and *p*-values are stated in the figure legends.

## Results

### Expansion of γδ T Cells From Healthy Donors

**Figure 1:**
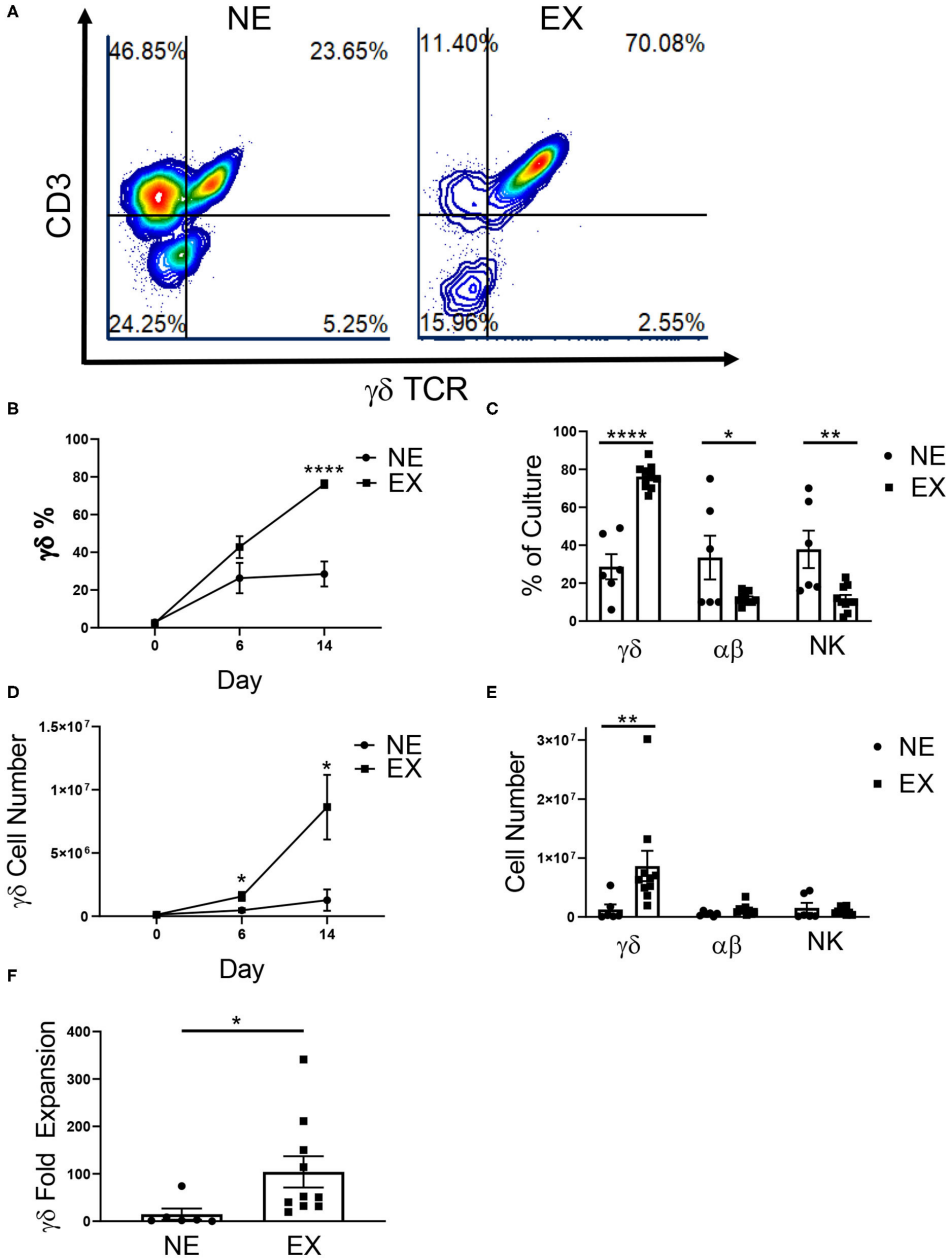
Expansion of healthy donor γδ T cells. γδ T cells were expanded from healthy donors, labeled either Non-Expanders (NE, *n* = 6) or Expanders (EX, *n* = 10) in two independent experiments. **(A)** Representative flow cytometry plots showing the percentage of γδ T cells (CD3+ γδTCR+), αβ T cells (CD3+ γδTCR–), and NK cells (CD3– γδTCR–CD56+) in culture on day 14 of expansion. **(B)** The percentage of γδ T cells was significantly higher for EX by day 14 of expansion (Student's *t-test, p* < 0.000001). **(C)** On day 14, EX had a greater percentage of γδ T cells in culture (*p* < 0.0001), while NE had a greater percentage of αβ T cells (*p* = 0.022) and NK cells (*p* = 0.003) (two-way ANOVA). **(D)** Compared to NEs, EXs had a larger number of γδ T cells in culture on day 6 (Student's *t-test, p* = 0.037) and 14 (Student's *t-test, p* = 0.048) of expansion. **(E)** EX had a greater number of γδ T cells compared to NE (*p* = 0.002), however there were no differences between EX and NE for the number of αβ T cells or NK cells in culture (two-way ANOVA). **(F)** Due to greater cell growth, EX also had a greater fold expansion of γδ T cells (Student's *t-test, p* = 0.031). *p < 0.05; **p < 0.005; ****p < 0.00005.

Variability in the expansion of γδ T cells from healthy donor PBMCs has been reported by multiple groups and poses a challenge in the development of immunotherapies utilizing γδ T cells. To characterize donor variability, donors were either classified as Non-Expanders (NE) or Expanders (EX) according to the percentage of γδ T cells in culture on the final day of expansion. Donors that had 60% or more γδ T cells on the final day of expansion were classified as EX, while donors that had <60% of γδ T cells were classified as NE (Figure 1A).

In this study, we examined 16 donors and demonstrated that 62.5% expanded (10 of the 16 donors). Interestingly, 100% of self-reported exercisers were classified as EX, while only 25% of sedentary donors were EX (Table 1). The relationship between these variables was significant (*p* = 0.007) such that γδ T cells from donors who exercise were more likely to expand. There was no difference in the starting percentage of γδ T cells in culture between NE (2.91% ± 0.75%) or EX (2.48% ± 0.52%) on day 0 of expansion. However, EX had a higher percentage (76.16% ± 2.05%) of γδ T cells in culture when compared to NE (28.47% ± 6.62%) on day 14 of expansion (Figure 1B). In contrast, when comparing αβ T cell and NK cell growth on day 14, NE had an average of 33.5% ± 10.54% αβ T cells and 37.83% ± 8.98% NK cells as compared to the 12.1% ± 0.93% αβ T cells and 11.7% ± 1.98% NK cells in EX cultures (Figure 1C).

**Table 1:** A chi-square analysis comparing the relationship between exercise and *ex vivo* γδ T cell expansion (*p* = 0.007).

	**Expander**	**Non-expander**
Exerciser	8	0
Sedentary	2	6

*Values indicate the number of donors in each category*.

Total cell growth and fold expansion was greater for EX (6.75 × 10^6^ ± 1.59 × 10^6^, 2.41-fold ± 0.63) compared to NE (3.28 × 10^6^ ± 3.67 × 10^5^, 0.74-fold ± 0.40) (Supplementary Figure 1). There was no difference in the starting number (NE = 1.30 × 10^5^ ± 3.38 × 10^4^, EX = 1.16 × 10^5^ ± 2.18 × 10^4^) of γδ T cells in culture on day 0 of expansion. However, EX had an average number of 1.57 × 10^6^ ± 3.50 × 10^5^ γδ T cells in culture by day 6 of expansion compared to 4.69 × 10^5^ ± 1.30 × 10^5^ γδ T cells for NE. Additionally, EX had an average number of 8.63 × 10^6^ ± 2.56 × 10^6^ γδ T cells in culture on day 14, while NE had an average of 1.27 × 10^6^ ± 8.44 × 10^5^ γδ T cells (Figure 1D). Although NE had a higher percentage of αβ T cells and NK cells on day 14, we found no significant differences in the numbers of αβ T cells and NK cells between NE (5.8 × 10^5^ ± 1.35 × 10^5^ and 1.53 × 10^6^ ± 7.85 × 10^5^) and E × (1.23 × 10^6^ ± 2.74 × 10^5^ and 9.92 × 10^5^ ± 1.87 × 10^5^) (Figure 1E). This difference in growth contributed to a γδ T cell fold expansion of 104.2-fold ± 32.84 for EX compared to 14.97-fold ± 11.93 for NE (Figure 1F).

To test the reproducibility of donor expansion across time, γδ T cells from two EXs were expanded from PBMCs isolated from two different blood draws. We found no significant differences in the percentage of γδ T cells or the number of γδ T cells in culture for either donor across the two time points (Supplementary Figure 2).

### Characterization of NE and EX at the End of Expansion

**Figure 2:**
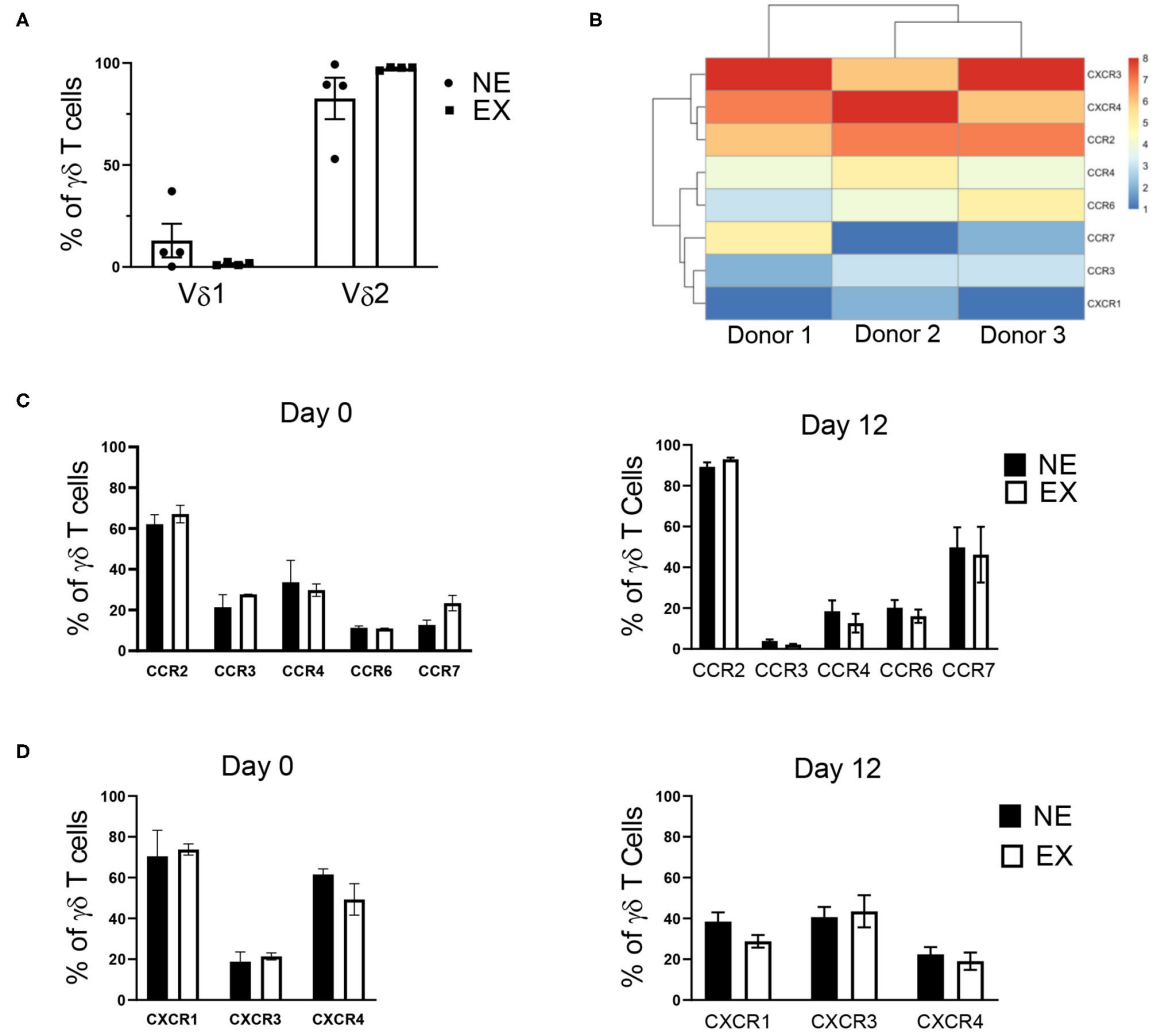
γδ T cell receptor subtype and chemokine receptor expression analyzed via flow cytometry and RNA-sequencing. **(A)** The majority of γδ T cells expanded from both NE (*n* = 4) and EX (*n* = 4) were of the Vδ2 subset. **(B)** RNA-sequencing revealed that CCR2, CXCR3, and CXCR4 had the highest expression of the chemokine receptors expressed on γδ T cells at the end of expansion. **(C)** There was no difference in CC receptor expression on γδ T cells from NE or EX on day 0 or day 12 of expansion. Of the CC chemokine receptors, CCR2 was highly expressed, while CCR4, CCR6, and CCR7 were only moderately expressed. CCR3 had the lowest expression of any chemokine receptors evaluated. **(D)** CXCR1, CXCR2, and CXCR4 were expressed at moderate levels on γδ T cells from both NE and EX on both day 0 and day 12 of expansion.

Flow cytometry was used to characterize Vδ receptor subset type and the expression of chemokine receptors, which are influential in the ability of T cell trafficking toward tumors. In evaluating γδ T cells for Vδ receptor subset and the expression of chemokine receptors, γδ T cells expanded from both NE and EX were primarily composed of the Vδ2 subtype, rather than Vδ1 (Figure 2A). Based on RNA-seq data from 3 EX donors (Figure 2B), 5 CC and 4 CXC chemokine receptors were chosen for further evaluation by flow cytometry. There was no difference in the baseline expression of chemokine receptors on day 0 of expansion between NE and EX (Figures 2C,D). Of the chemokine receptors we analyzed on day 12 of expansion, CCR2 had the highest expression on γδ T cells from both NE (89.28% ± 2.19%) and EX (92.92% ± 0.83%). CCR4, CCR6, CCR7, CXCR1, CXCR3, and CXCR4 were all moderately expressed at similar levels on NE and EX γδ T cells (Figures 2C,D). CCR3 was expressed similarly on γδ T cells from NE (3.89% ± 2.19%) and EX (2.14% ± 0.29%), although it had the lowest expression of the receptors tested (Figure 2D).

### IL-21 Increases the Percentage and Number of γδ T Cells From NE

**Figure 3:**
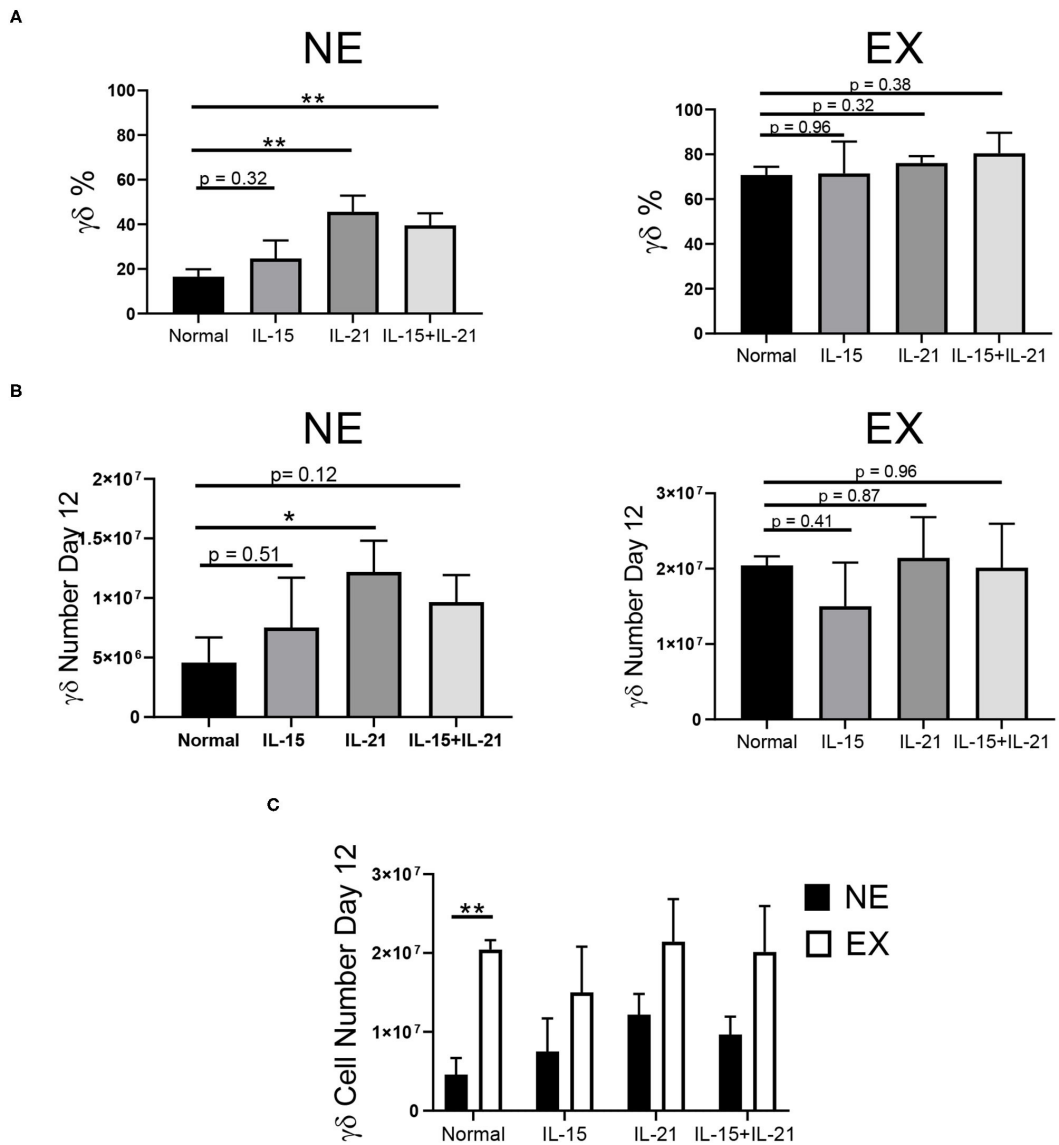
Effects of IL-15, IL-21, and IL-15+IL-21 on expansion across three independent experiments. **(A)** The addition of IL-21 (Student's *t-test, p* = 0.0027) and IL-15+IL-21 (Student's *t-test, p* = 0.0035) increased γδ T cell percentage by day 12 of NE (*n* = 7) in comparison to normal expansion conditions. Adding IL-15, IL-21, and IL-15+IL-21 had no effect on the percentage of γδ T cells in culture on day 12 for EX (*n* = 3) (Student's *t-test, p* > 0.05). **(B)** The number of γδ T cells in culture on day 12 for NE was increased with the addition of IL-21 (Student's *t-test, p* = 0.0427), but not for IL-15 or IL-15+IL-21 (multiple Student's *t-test*s, *p* > 0.05). There was no difference in the number of γδ T cells in culture on day 12 for EX under normal expansion conditions or with the addition of IL-15, IL-21, or IL-15+IL-21 (multiple Student's *t-test*s, *p* > 0.05). **(C)** EX had a greater number of γδ T cells on day 12 under normal expansion conditions as compared to NE (Student's *t-test, p* = 0.002), however there was no difference in the number of γδ T cells in culture on day 12 between NE and EX with the addition of IL-15, IL-21, and IL-15+IL-21 (multiple Student's *t-test*s, *p* > 0.05). *p < 0.05; **p < 0.005.

Common gamma chain cytokines, including IL-2, IL-7, IL-15, and IL-21, have been implicated in the expansion of T cells. Therefore, we investigated the use of these cytokines in combination with IL-2 to determine if they could provide benefit in the expansion of γδ T cells. The effects of IL-7, IL-15, IL-21, and IL-15 + IL-21 on the expansion of γδ T cells was determined for NE and EX. IL-7 decreased the expansion of γδ T cells for both EX and NE and was not investigated further (data not shown). IL-15 had no effect on the percentage or number of γδ T cells in culture for NE. The addition of IL-21 into the culture increased the percentage and number of γδ T cells in culture for NE by day 12 of expansion (Figures 3A,B, Supplementary Figure 3). Similarly, the combination of IL-15 + IL-21 also increased the percentage of γδ T cells in culture for NE by day 12 and had a trend toward increased γδ T cell number. The addition of IL-15, IL-21, and IL-15 + IL-21 had no effect on the γδ T cell percentage for EX. However, as shown in Figure 3C, EX had significantly more γδ T cells on day 12 of expansion under normal conditions as compared to NE. Although there was a trend toward fewer cell numbers overall for NE as compared to EX, the addition of IL-15, IL-21, and IL-15 + IL-21 increased NE γδ T cell numbers to levels similar to those of EX.

The use of IL-21 in the expansion of γδ T cells has been associated with a reduction in cytotoxic capacity (41). Therefore, we characterized the γδ T cells expanded with IL-21 to ensure that it had no negative impact on functionality or phenotype. The addition of IL-21 into our expansion conditions had no impact on the activation of γδ T cells for NE or EX (Supplementary Figure 4A), as assessed by the expression of CD69. Similarly, there was no effect of IL-21 on the cytotoxicity or degranulation of γδ T cells when incubated with K562 cells (Supplementary Figures 4B,C). Additionally, there was no difference in expression of exhaustion (PD-1, TIM3, CD244, and CTLA-4) or senescence (KLRG1 and CD85j) markers on day 12 for cultures expanded under normal conditions or with the addition of IL-21 (Supplementary Figure 4D).

### αβ Depletion During Expansion

To determine if αβ depletions could be performed early in the expansion process, γδ T cells were αβ-depleted on day 0, 3, 6, and 9 of expansion. As observed in Supplementary Figure 1A, cell growth peaks at day 12 of expansion and because of this, day 12 was used as the endpoint analysis for all further studies. Depleting αβ T cells on day 0 or 3 resulted in a significant loss of γδ T cells, and due to low initial numbers of γδ T cells was not evaluated further (data not shown). Cultures depleted of αβ T cells on day 6 had a lower percentage of γδ T cells post-depletion (75.34% ± 5.88%) compared to the day 9 depleted cultures (91.69% ± 1.53%) due to a larger starting population of NK cells. Similar to reports characterizing the efficiency of αβ T cell depletion, we report a recovery of γδ T cells between 47 and 77% (Supplementary Figure 5A). Of the γδ T cells lost during the αβ depletion procedure, a percentage was recovered in the αβ fraction (Supplementary Figure 5B), and these cells could also be expanded, although not to cell numbers relevant for therapeutic use (Supplementary Figure 5C).

**Figure 4:**
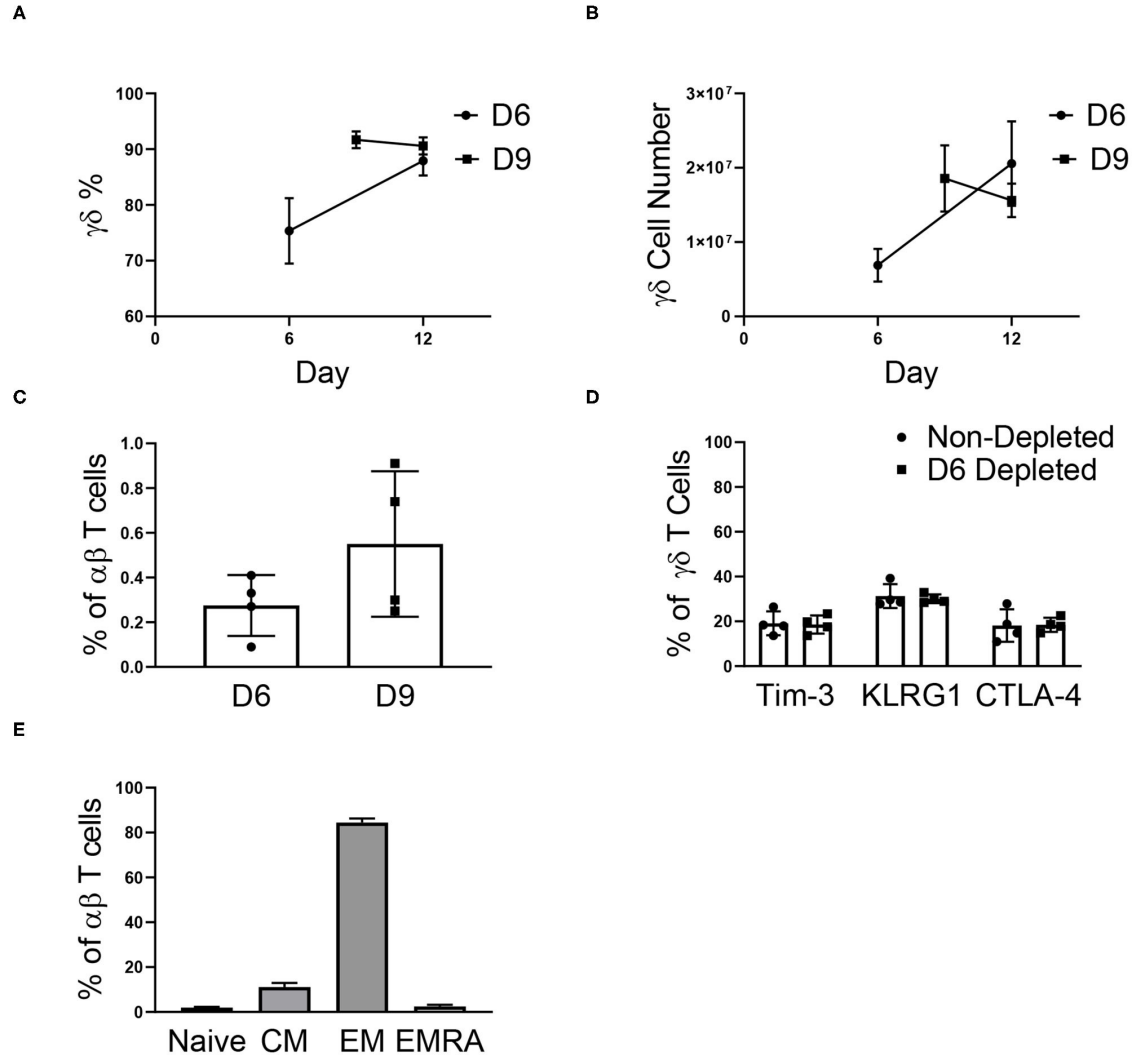
αβ depletions performed on day 6 (D6) and 9 (D9) of expansion (two independent experiments). **(A)** The percentage of γδ T cells post-depletion was lower when the depletion was performed on day 6 (*n* = 6) of expansion vs. day 9 (*n* = 6) of expansion, however by day 12 there was no difference in the percent of γδ T cells in culture. **(B)** The number of γδ T cells in culture post-depletion to the end of expansion followed the same trend as the percentage of γδ T cells in culture. **(C)** Following depletion, αβ T cells made up <1% of the culture. **(D)** Performing the αβ depletion did not induce senescence in the γδ T cells by the end of expansion, as compared to a non-depleted culture. **(E)** Flow cytometry was performed determine the proportion of naïve (CD27+CD45RA+), central memory (CD27+CD45RA–), effector memory (CD27–CD45RA–), and effector memory CD45RA+ (CD27–CD45RA+) αβ T cells in culture. Of the αβ T cells in culture on day 12, the majority of cells were of the EM phenotype.

For cultures depleted on day 6, the percentage of γδ T cells increased through day 12, and by the end of expansion, we found no difference in the percentage of γδ T cells for day 6 (87.93% ± 2.63%) and day 9 (90.59% ± 1.54%) depleted cultures (Figure 4A). Following αβ T cell depletion, γδ T cells continued to expand, regardless of the day of depletion (Figure 4B). Although the depletions were performed on day 6 or day 9 of expansion, the cultures remained depleted through day 12, with <1% αβ T cells in culture (Figure 4C). Additionally, the depletion process did not induce an up-regulation of senescent markers on the γδ T cells, as compared to non-depleted cultures (Figure 4D).

Although <1% of our expanded γδ T cell cultures are typically comprised of αβ T cells, the phenotype of the remaining αβ T cells has important implications for graft vs. host disease (GVHD) risk. Therefore, the remaining αβ T cell phenotypes were characterized in culture (Figure 4E). Of the αβ T cells in culture, 84.44% ± 1.81% were effector memory (EM) cells, 2.49% ± 0.76% were effector memory CD45RA+ (EMRA) cells, 11.15% ± 1.79% were central memory cells (CM), and 1.93% ± 0.36% were naïve cells.

### Mixed Donor γδ T Cell Immunotherapy

**Figure 5:**
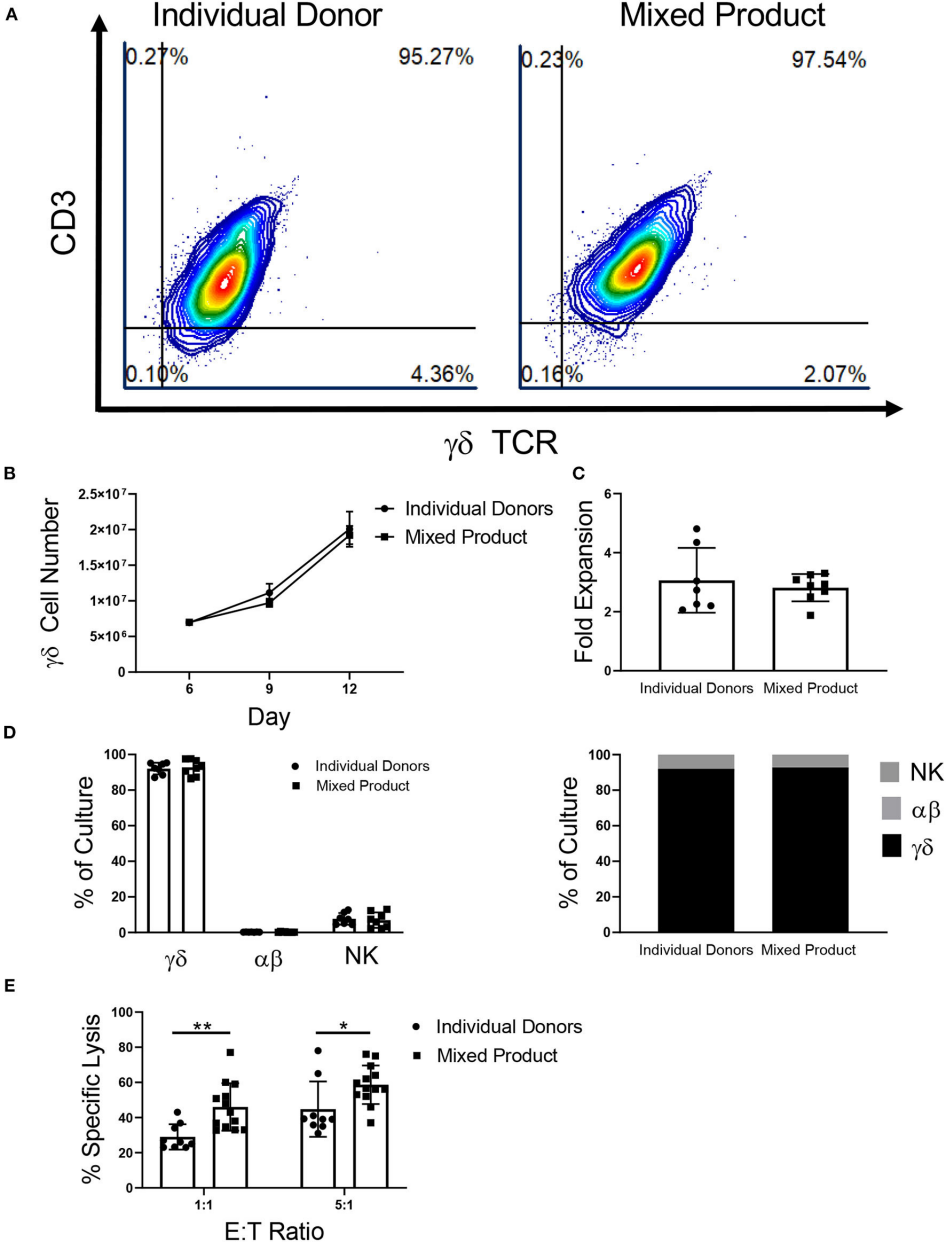
Characterization of a mixed γδ T cell product. **(A)** Representative flow cytometry plots showing that γδ T cells from individual donors and the mixed product expand to a uniform cellular product by day 12 of expansion (two independent experiments). **(B)** There was no difference in γδ T cell growth between γδ T cells from the individual donors (*n* = 7) and the mixed product (*n* = 8) between day 6 and day 12 of expansion (two-way ANOVA). **(C)** No difference was seen in the fold expansion of individual donor γδ T cells or the mixed product between day 6 and day 12 of expansion (Student's *t-test, p* > 0.05). **(D)** On day 12 of expansion, there was no difference in the percentage of γδ T cells, αβ T cells, or NK cells in the cultures from individual donors and the mixed product (two-way ANOVA, *p* > 0.05). **(E)** Cytotoxicity of the mixed cell product against K562 cells was greater at the 1:1 (*p* = 0.006) and 5:1 (*p* = 0.03) ratios when compared to the cytotoxicity of individual donor γδ T cells (two-way ANOVA).

γδ T cells provide an opportunity for a mixed donor T cell immunotherapy because of their low risk for GVHD and cross-sample cytotoxicity due to the lack of HLA-γδ TCR engagement. The aim of these experiments was to determine if γδ T cells from different donors could be mixed and expanded in culture together to create a uniform cellular product. After αβ T cell depletion on day 6, the γδ T cells from three individual donors were mixed. The percentage of γδ T cells in the mixed donor γδ T cell product remained constant throughout expansion (Figure 5A). In contrast, when the αβ fractions from three separate donors were mixed post-depletion (on day 6), three distinct products were observed in the expansion (Supplementary Figure 6A). The γδ T cell mixed product continued to grow throughout day 12 (Figure 5B), with an average fold expansion of 2.81-fold ± 0.16 after mixing (Figure 5C), while the αβ mixed samples had only a small increase in fold expansion (1.10-fold ± 0.21; Supplementary Figures 6B,C).

Similar to γδ T cells expanded from individual donors, the mixed product was comprised of 92.78% ± 1.57% γδ T cells, 0.27% ± 0.06% αβ T cells, and 6.96% ± 1.52% NK cells on day 12 of expansion (Figure 5D). Moreover, the individual donors used for the mixed cell product expanded to a final composition made up of 92.07% ± 1.24% γδ T cells, 0.23% ± 0.02% αβ T cells, and 7.69% ± 1.25% NK cells. Comparing the mixed γδ T cell product to individual donor γδ T cells, we observed increased cytotoxicity toward K562 myeloid leukemia cells at a ratio of 1:1 and 5:1 (Figure 5E).

### Cyropreservation of the γδ T Cell Product

**Figure 6:**
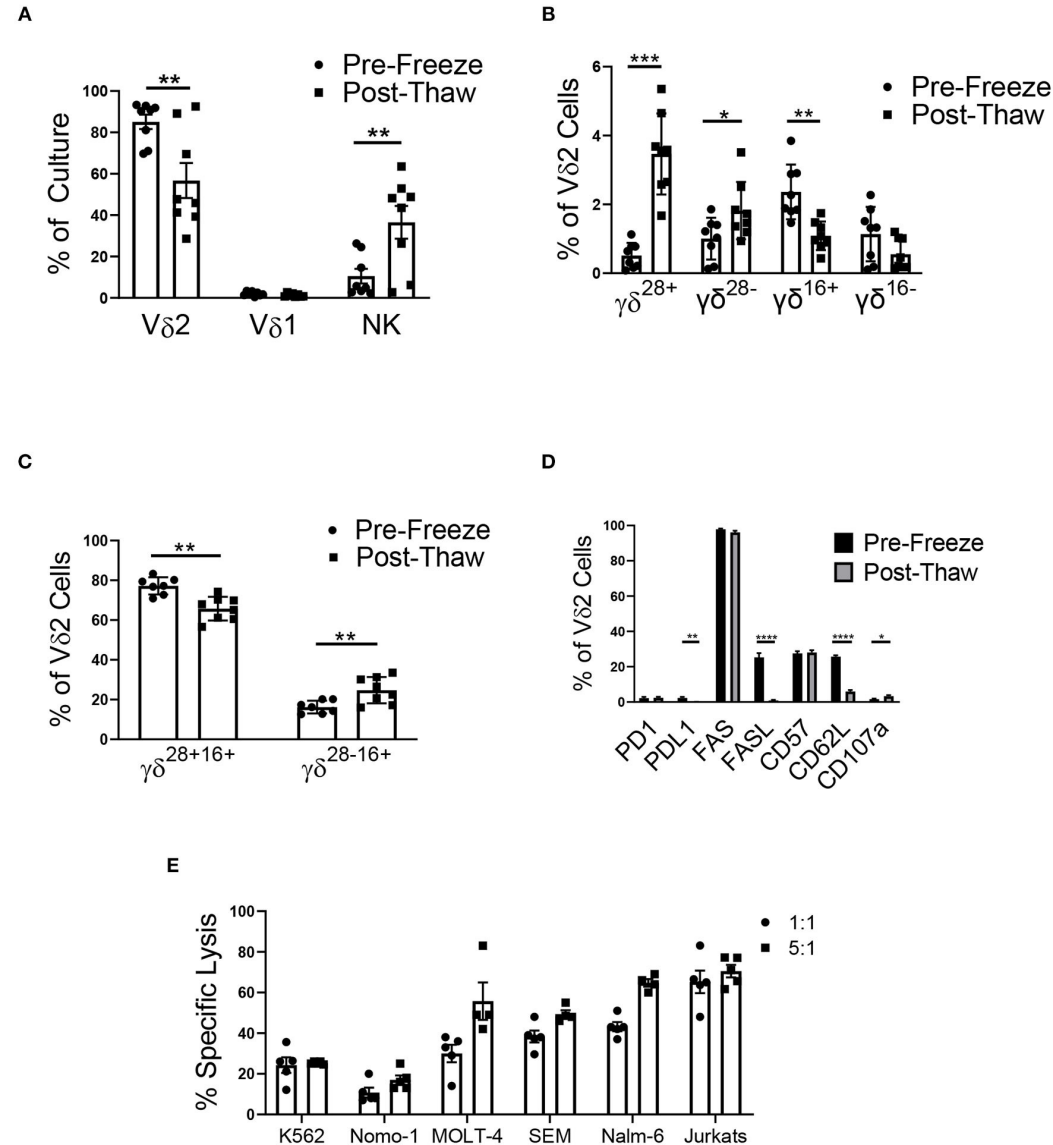
Cryopreservation of the mixed cell product (*n* = 8, two independent experiments). **(A)** The mixed cell product experienced a decrease in the percentage of Vδ2 γδ T cells post-thaw (Student's *t-test, p* = 0.008), while there was no change in the percentage of Vδ1 γδ T cells (Student's *t-test, p* > 0.05), and there was an increase in the percentage of NK cells (Student's *t-test, p* = 0.01). **(B)** γδ T cells were categorized into four phenotypes using CD28, CD27, and CD16: γδ^28+^ (CD28+CD27+CD16–), γδ^28−^ (CD28–CD27+CD16–), γδ^16+^ (CD28–CD27–CD16+), and γδ^16−^ (CD28–CD27–CD16–). There was no difference in the percentage of γδ^16−^ Vδ2 γδ T cells post-thaw. The percentage of γδ^28+^ cells (Student's *t-test, p* = 0.0005) and γδ^28−^ (Student's *t-test, p* = 0.04) increased post-thaw, while the percentage of γδ^16+^ decreased (Student's *t-test, p* = 0.009). **(C)** To account for the bulk population of γδ T cells, the Vδ2 cells were also categorized into γδ^28+16+^ (CD28+CD27+CD16+) and γδ^28−16+^ (CD28–CD27+CD16+). There was a decrease in the percentage of γδ^28+16+^ cells post-thaw (Student's *t-test, p* = 0.000009), while the percentage of γδ^28−16+^ cells increased (Student's *t-test, p* = 0.0013). **(D)** The percent of Vδ2 cells that expressed PD1, FAS, and CD57 did not change after thawing. The percent of PDL1+ (Student's *t-test, p* = 0.001), FASL+ (Student's *t-test, p* < 0.000001), and CD62L+ (Student's *t-test, p* < 0.000001) Vδ2 cells increased post-thaw, while the percent of CD107a+ cells increased (Student's *t-test, p* = 0.03). **(E)** Cytotoxicity of the mixed γδ T cell product 48 h post-thaw against a range of leukemia cell lines: K562, Nomo-1, Molt-4, SEM, Nalm-6, and Jurkats. *p < 0.05; **p < 0.005; ***p < 0.0005; ****p < 0.00005.

After cryopreservation, there was a significant decrease in the percentage of Vδ2 γδ T cells in culture (Figure 6A). Vδ1 γδ T cells made up <4% of all γδ T cells in culture, and cryopreservation had no effect on this percentage. The percentage of NK cells significantly increased post-thaw, likely due to the decrease in total viable Vδ2 γδ T cells.

Phenotypic populations of γδ T cells are difficult to differentiate using the common markers, CD27 and CD45RA. Instead, a recent report has shown that γδ T cells can be differentiated into four main phenotypes using CD27, CD28, and CD16: γδ^28+^ (CD28+CD27+CD16-), γδ^28−^ (CD28-CD27+CD16-), γδ^16+^ (CD28-CD27-CD16+), and γδ^16−^ (CD28-CD27-CD16-), with γδ^28+^ being the most common phenotype. In assessing the surface marker (CD27, CD28, and CD16) phenotypes of our mixed donor cell product before and after cryopreservation, we demonstrated an increase in the percentage of γδ^28+^ and γδ^28−^ Vδ2 cells, a decrease in γδ^16+^ Vδ2 cells, and no change in the percentage of γδ^16−^ cells (Figure 6B). Of note, these classifications made up no more than 7% of our mixed product, and as a result, we characterized the remaining bulk population of γδ T cells into γδ^28+16+^ (CD28+CD27+CD16+) and γδ^28−16+^ (CD28–CD27+CD16+) to account for the majority of the cell population. The γδ^28+16+^ phenotype comprised 77.2 ± 1.5% of the Vδ2 population, which decreased to 65.7 ± 2.1% post-thaw. The γδ^28−16+^ population increased from 16.2 ± 1.1% pre-freeze to 24.7 ± 2.3% post-thaw (Figure 6C). Cryopreservation had no effect on the PD1 or FAS expression on Vδ2 γδ T cells in the mixed product. However, expression of their ligands, PDL1 and FASL, was decreased post-thaw (Figure 6D), and CD62L and CD107a expression decreased post-thaw, while CD57 remained unchanged.

To ensure the mixed γδ T cell product retained functionality after undergoing a freeze/thaw cycle, the mixed product was tested in a cytotoxicity assay against a range of leukemia cell lines. After thawing, the cells were retained cytotoxicity at the 1:1 and 5:1 effector to target ratio against K562 (24.3% ± 3.8% and 25.3% ± 0.3%), Nomo-1 (10.8% ± 2.4% and 17% ± 2.2%), MOLT-4 (30.0% ± 4.3% and 55.8% ± 9.2%), SEM (38.4% ± 2.9% and 49.4% ± 2.0%), Nalm-6 (43.2% ± 2.2% and 64.8% ± 1.9%), and Jurkats (65.3% ± 5.7% and 70.5% ± 3.1%) cell lines (Figure 6E and Supplementary Figure 7).

## Discussion

Variation in the expansion of γδ T cells from different donors has been reported across the literature. Donors that expand are ideal candidates for the development of an allogeneic γδ T cell therapy, as they have a higher percentage and fold expansion of γδ T cells and therefore also provide a greater number of cells to use for product manufacturing. Additionally, we have shown that individual donor expansion is reproducible. In the context of an allogeneic therapy, having donors fail to expand can delay treatment and increase the cost of production for the therapy. To better define the starting cell population, we analyzed the starting percentage of γδ T cells for NE and EX and found no difference, suggesting that the percentage of circulating γδ T cells in the peripheral blood cannot be used as a predictor for the success of a donor's expansion.

Treating PBMCs with zoledronate and IL-2 results in the selective expansion of γδ T cells. αβ T cells and NK cells also grow in a donor-dependent manner. Our data show that γδ T cells from EX grow preferentially over αβ T cells and NK cells in our specified culture conditions. In contrast, we find no preferential expansion of γδ T cells in a culture of PBMCs from donors that are NE. In fact, of the six NE in this study, half of the cultures were primarily comprised of αβ T cells at the end of expansion, while the other half were primarily comprised of NK cells. It is possible that the donor variability observed in this study, as well as others, could be accounted for by variability in the donors' lifestyles. Exercise immediately prior to PBMC isolation has been shown to increase the *ex vivo* expansion of γδ T cells (42). In this study, a donor's level of exercise was predictive of their γδ T cell expansion potential. While 100% of donors who reported high levels of exercise were expanders, only 25% of sedentary donors were classified as expanders. Taken together, these studies suggest that a donor's level of physical activity can be indicative of whether or not their γδ T cells will expand *ex vivo*. To better understand the difference in expansion potential of NE and EX, further studies characterizing the starting cellular populations are necessary. RNA-sequencing performed immediately after isolation from the blood, paired with expansion data, could give insight into a marker that could predict whether γδ T cells from a particular donor will expand.

There is substantial variation in cell culture methods used to expand γδ T cells. Variation can be found in the media (RPMI, IMDEM, OpTmizer), the addition or absence of serum (FBS, human AB serum), and the type of stimulatory molecules used (i.e., phosphoantigens, aminobisphosphonates, and cytokines). We chose to use the serum-free protocol developed by our laboratory, which uses a combination of zoledronate and IL-2. It is well-known that IL-2 is beneficial for the selective expansion of γδ T cells *ex vivo*. Additional cytokines have also been implicated in the expansion of γδ T cells. In this study, we investigated common gamma chain cytokines that have a role in the expansion of T cells: IL-7, IL-15, and IL-21 (43–45). IL-7 decreased the expansion of γδ T cells (data not shown) and was not investigated further. Van Acker et al. reported successful γδ T cell expansion with the addition of IL-15 (45), however, we found that it had no benefit in our culture conditions for either NE or EX. These conflicting results could be due to differences in the expansion protocol used. Our studies are unique in that we assess the benefits of common gamma chain cytokines under serum-free conditions, which is more clinically relevant compared to serum-containing protocols.

The addition of IL-21 increased the expansion of γδ T cells from NE, but had no effect on the expansion of γδ T cells from EX. IL-21 has been shown to induce the proliferation of natural killer (NK) cells and increase proliferation of activated T cells (46). Vermijlen et al. showed that IL-21 increased γδ T cell expansion, although not to levels greater than expansion induced with IL-2 (47). Additionally, it is known that IL-21 can enhance the effects of IL-2 and IL-15 on T cell proliferation, which might explain the increase in γδ T cell percentage observed in the IL-15 plus IL-21 condition for NE. These results are significant in the context of adoptive cell therapies in which a patient must receive γδ T cells from HLA-matched donors. Being able to increase the *ex vivo* expansion of NE γδ T cells by the addition of a cytokine would suggest that a successful γδ T cell therapy could be produced from any donor, not just an EX.

αβ T cell depletions are a necessary step in the development of an allogeneic γδ T cell immunotherapy due to the risk for graft vs. host disease (GVHD), which is initiated by naïve αβ T cells (48, 49). Typically, αβ T cell depletions are performed at or near the end of γδ T cell expansions. However, our aim was to successfully deplete αβ T cells in an EX culture at an earlier time point. Here, we have demonstrated that αβ T cells can be depleted on day 6 or day 9 of expansion and that the culture remains depleted of αβ T cells through day 12. Most importantly, αβ T cells accounted for <1% of the total cell population on day 12 and of those that remain, <2% were naïve cells. This shows the αβ T cells remaining in culture present an extremely low risk for GVHD (50). Depleting the αβ T cells in the middle of expansion, compared to the end, requires the use of fewer reagents, which is practical in the context of creating a viable and cost effective cell therapy. Additionally, for the donors tested in this study, depleting the αβ T cells earlier in the expansion resulted in a reduction in the NK cell population by day 12, as compared to the non-depleted cultures.

Creating a mixed donor γδ T cell product is a novel approach toward improving γδ T cell therapies. Due to variability in the expansion and cytotoxicity of γδ T cells from different donors, a mixed donor cell product provides an opportunity to increase the therapeutic efficacy of γδ T cell cancer immunotherapies. Unlike αβ T cells, the risk of developing GVHD is extremely low for patients treated with γδ T cells, making a cell product combined from different donors feasible. We report that γδ T cells from multiple donors can be mixed after αβ T cell depletion and successfully expanded to create a uniform cellular product comprised of 93% γδ T cells and 7% NK cells, on average. When compared to γδ T cells from individual donors, the mixed product had greater cytotoxicity toward leukemia cells *in vitro*. These results suggest that γδ T cells mixed from different donors may have a synergistic effect on each other, resulting in a cell product with greater overall cytotoxicity. Although NK cells comprised 7% of the mixed product, depletion was not considered because NK cells also present low risk for GVHD, are cytotoxic against a range of cancers, and may contribute to the cytotoxicity seen in the γδ cell product.

Cryopreservation is a necessary step in the translation of an off-the-shelf cell product and often requires optimization. Limited information is known about the cryopreservation of γδ T cells and the effects of a freeze/thaw cycle on their health. We characterized the composition of the cell product before and after freezing to determine if there were any phenotypic changes associated with cryopreservation. In the present work, the composition of the mixed product changed dramatically after thawing, with a significant reduction in the percent of Vδ2 γδ T cells and an increase in the percent of NK cells. A recent study extensively characterized the phenotype of γδ T cells based on CD28, CD27, and CD16 expression (51). This group found that there were four main phenotypes that could be used to classify γδ T cells: γδ^28+^, γδ^28−^, γδ^16+^, and γδ^16−^. However, these four phenotypes could only be used to classify up to 7% of our mixed cell product and we further characterized our cell product into two additional phenotypes: γδ^28+16+^ and γδ^28−^. Ryan et al. found that CD27 expression on γδ T cells was an indicator of expansion potential, while CD16 expression was an indicator of higher levels of cytotoxicity (41). Over 93% of γδ T cells in the mixed product were CD27+CD16+, which could account for their high levels of expansion and cytotoxicity. Additionally, high expression of CD27 on these γδ T cells is important in determining the role that they could have in promoting tumor progression. Studies have shown that a subset of γδ T cells, specifically those that produce IL-17, play a role in tumor progression. IL-17 producing γδ T cells either do not express CD27 or express low levels of CD27 (52), suggesting that γδ T cells expanded using this protocol will not promote tumor progression *in vivo*.

After thawing, the final mixed product was comprised of an average of 66% γδ^28+16+^ and 25% γδ^28−16+^ γδ T cells, which is similar to levels our laboratory has previously published on γδ T cells expanded from neuroblastoma patient-derived apheresis products (27). Cryopreservation significantly decreased the population of γδ^28+16+^ Vδ2 γδ T cells and increased the population of γδ^28−16+^ cells. As the loss of CD28 on T cells can be an indicator of senescence (53), the increase in γδ^28−16+^ cells suggests that cryopreservation increases levels of senescence in γδ T cells after thawing. However, the decreased expression of PDL1 and FASL suggests that the mixed product may be less susceptible to tumor-induced apoptosis (54) and activation-induced cell death (55) after a freeze/thaw cycle. Although we show that cryopreserved γδ T cells were cytotoxic against a range of leukemia cell lines, the overall cytotoxicity of the mixed product against K562 cells before and after freezing was reduced. These results indicate that cryopreservation impacts the composition, phenotype, and functionality of γδ T cells and further optimization is necessary to reduce the harmful effects of the cryopreservation process.

In addition to optimizing the negative impacts of cryopreservation, an important consideration to optimize the health of the mixed γδ T-cell product is the timing with which the cells will be administered to patients during a regimen of chemotherapy. Chemotherapy negatively impacts the cytotoxicity of resident γδ T cells (56), suggesting that it could also negatively impact cells being infused into a patient if the two treatments are administered closely together. Further studies will be necessary to determine the impacts of chemotherapy on the mixed γδ T cell product and to optimize the course of treatment.

The characterization of the expansion of healthy donor γδ T cells from NE and EX is informative because it shows that the success of an expansion cannot be predicted based upon initial γδ T cell percentages. Instead, further research is necessary to determine if there are differences between γδ T cells from NE and EX after isolation from a donor. In the autologous cell transplant setting, where cells from cancer patients usually do not expand well, the use of IL-21 can “rescue” the expansion so that enough cells can be manufactured for treatment. Depleting αβ T cells during the expansion of γδ T cells greatly reduces the amount of reagents necessary for this procedure, allowing for a more cost-effective therapy that can be easily scaled up to clinical levels. Additionally, αβ T cell depletion during expansion allows for the development of a novel allogeneic mixed donor γδ T cell immunotherapy. We show here that a mixed donor γδ T cell immunotherapy has increased cytotoxicity in comparison to an individual donor γδ T cell immunotherapy. As clinical trials with γδ T cell immunotherapies have had limited efficacy, the mixed donor cell product should be considered for development as a more effective treatment.

## Data Availability Statement

The raw data supporting the conclusions of this article will be made available by the authors, without undue reservation.

## Author Contributions

REB designed research, performed research, collected data, analyzed data, and wrote the manuscript. JTZ, JYS, SNG, GG, AR, EW, LB, and DA performed research and analyzed data. CBD and HTS conceived and designed the research, analyzed data, and edited the manuscript. All authors reviewed the manuscript.

## Conflict of Interest

The authors declare that the research was conducted in the absence of any commercial or financial relationships that could be construed as a potential conflict of interest.
