# Supplementary material for: Characterization of Donor Variability for γδ T Cell ex vivo Expansion and Development of an Allogeneic γδ T Cell Immunotherapy
Source: Front Med (Lausanne). 2020 Nov 13;7:588453. doi: 10.3389/fmed.2020.588453 (PMC7691424; doi:10.3389/fmed.2020.588453)

**Supplemental Figures**

Supplemental Figure 1. Overall cell growth was evaluated for NE (n=6) and EX (n=10) in 2 independent experiments. A) The total cell growth was greater for EX on day 12 (p= 0.0028) and 14 (p= 0.0034) of expansion, when compared to NE (two-way ANOVA). B) EX also had a greater fold expansion as compared to NE (Student's *t* test, p= 0.039).

Supplemental Figure 2. To test the reproducibility of donor expansion across time, two EX were expanded at two different time points (2 independent experiments). A) There was no difference between the two expansions in the percentage of γδ T cells for either donor (paired Student's *t* test, $p > 0.05$). B) Similarly, there was no difference in the number of γδ T cells expanded from either donor at the two time points (paired Student's *t* test, $p > 0.05$).

Supplemental Figure 3. γδ T cell growth and percentage over time with the supplementation of IL-15, IL-21, or IL-15 and IL-21 in the culture conditions across 3 independent experiments. A) The percentage of γδ T cells in culture for NE and EX on day 0, 6, and 12 of expansion. B) γδ T cell growth for NE and EX. C) There was no difference in the starting percentage of γδ T cells between NE or EX (p= 0.56). D) The number of γδ T cells in culture on day 0 was not statistically significant between NE and EX (p= 0.51).

Supplemental Figure 4. Characterization of the activation, cytotoxicity, degranulation, exhaustion and senescence for γδ T cells expanded with IL-21 supplementation for NE (n=2) and EX (n=2) (1 independent experiment). Activation, cytotoxicity, and degranulation were assessed on day 12 of expansion after γδ T cells were incubated with K562 cells at a 1:1 ratio (activation, cytotoxicity, degranulation) or a 5:1 ratio (cytotoxicity) for 4 hours. A) There was no difference in CD69 expression for cultures expanded with and without IL-21 for either NE or EX. B) Supplementation

of IL-21 in the expansion conditions had no impact on cytotoxicity against K562 cells for either NE or EX. C) Similarly, IL-21 supplementation did not affect degranulation when γδ T cells were incubated with K562 cells for 4 hours. D) γδ T cells expanded with Il-21 did not have increased expression of exhaustion (PD-1, TIM3, CD244, CTLA-4) or senescence (KLRG1, CD85j) markers on day 12 as compared to cultures expanded under normal conditions.

Supplemental Figure 5. Analysis of the αβ depletion process. A) After the αβ depletion was performed, between 47-77% of the starting number of γδ T cells was recovered. B) A representative flow cytometry plot showing that some γδ T cells were lost to the αβ fraction after depletion. C) Representative flow cytometry plots showing that when the αβ fractions are cultured post-depletion, two phenotypes emerge. Phenotype 1 is primarily comprised of αβ T cells, while Phenotype 2 is primarily comprised of γδ T cells.

Supplemental Figure 6. Mixture of the αβ fraction post-depletion does not create a uniform cell product (n= 3, 1 independent experiment). A) Mixture of the αβ fraction after depletion from three donors resulted in three distinct cell products by day 12 of expansion. B) The total cell growth of the αβ mixed products decreased directly after mixing, but recovered to the initial starting number by day 12. C) Two of the three αβ mixed products had a small increase in the fold expansion between day 6 and day 12 of expansion, while the third had a decrease in fold expansion.

Supplemental Figure 7. Raw cytotoxicity data showing the background cell death of each negative control (0:1) for the K562, Nomo-1, Molt-4, SEM, Nalm-6, and Jurkats cell lines. Data graphed at the 1:1 and 5:1 effector:target ratios represents the raw cytotoxicity data before the background cell death was subtracted out. One-way ANOVAs were performed for each cell line to determine if target cell death was statistically significant at the 1:1 and 5:1 ratios as compared to the negative controls.

Supplemental Figure 1.

A

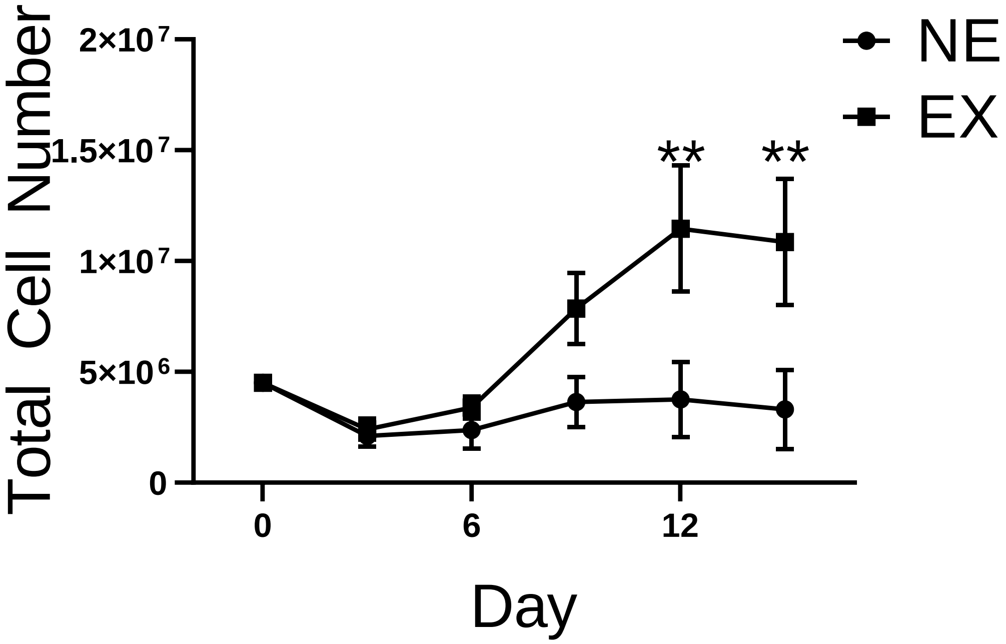


B

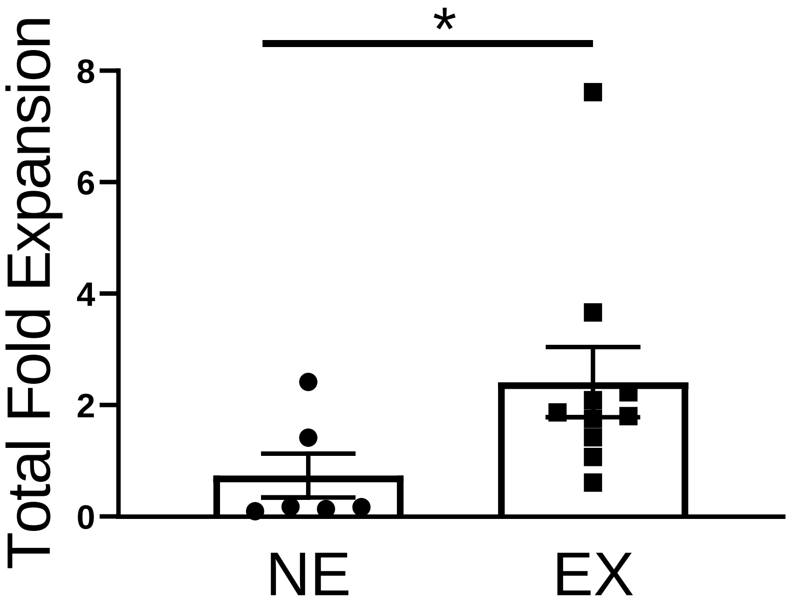

Supplemental Figure 2.

A

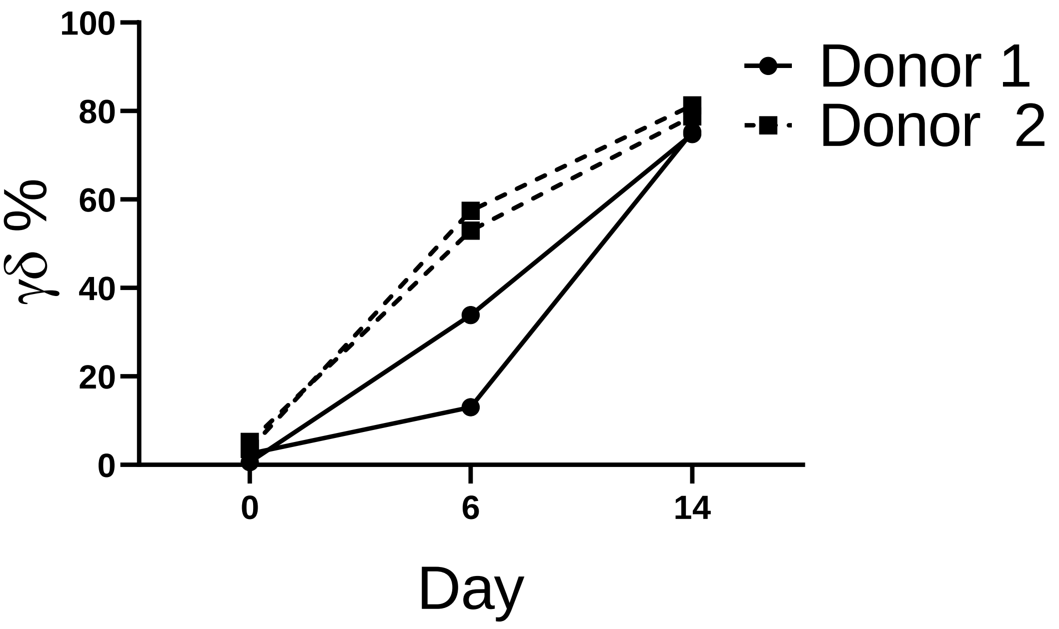


B

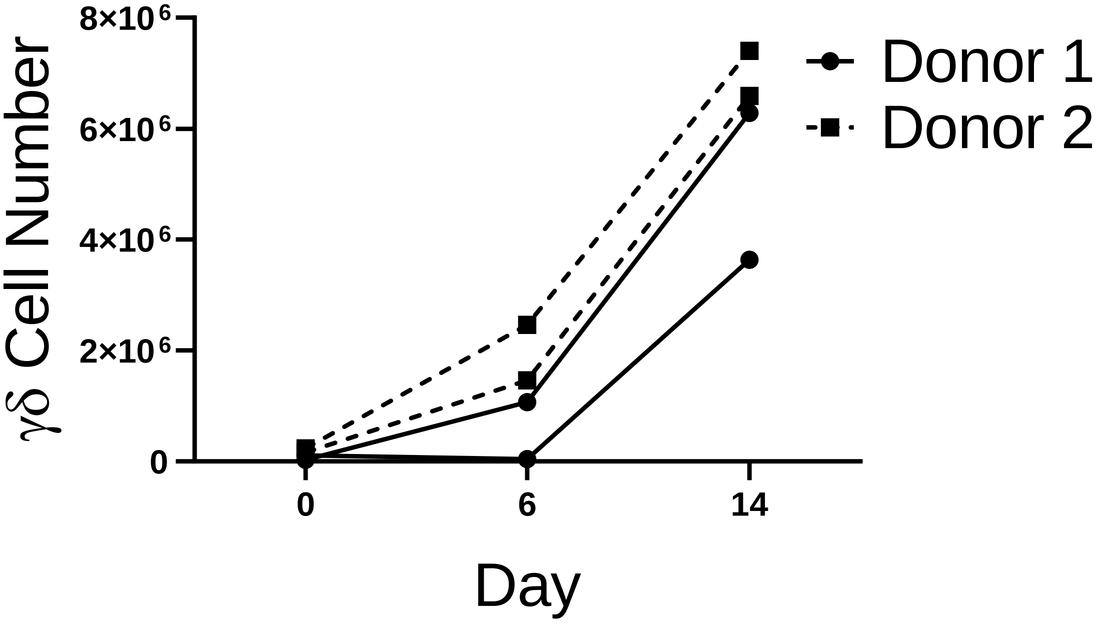

Supplemental Figure 3.

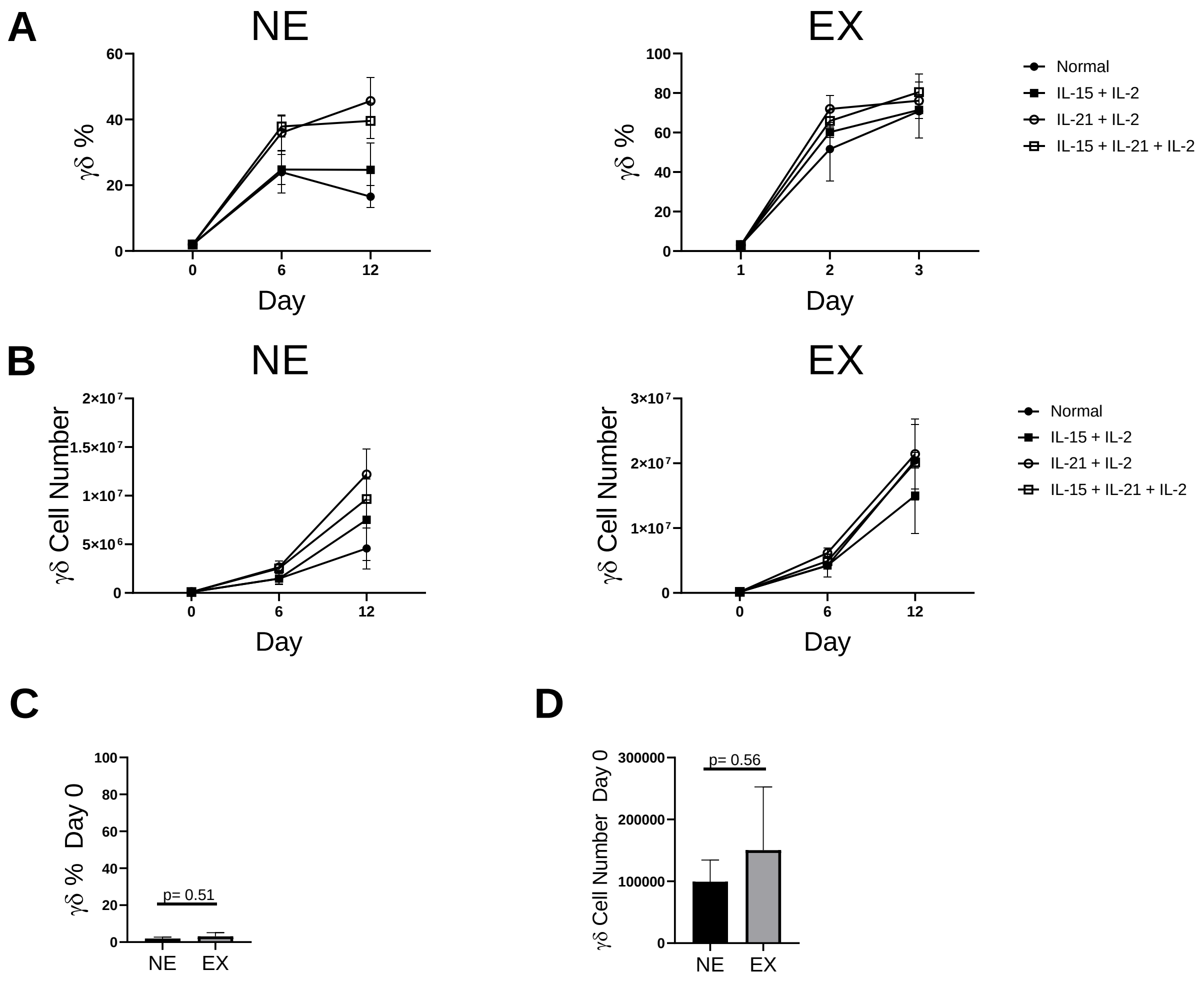

Supplemental Figure 4.

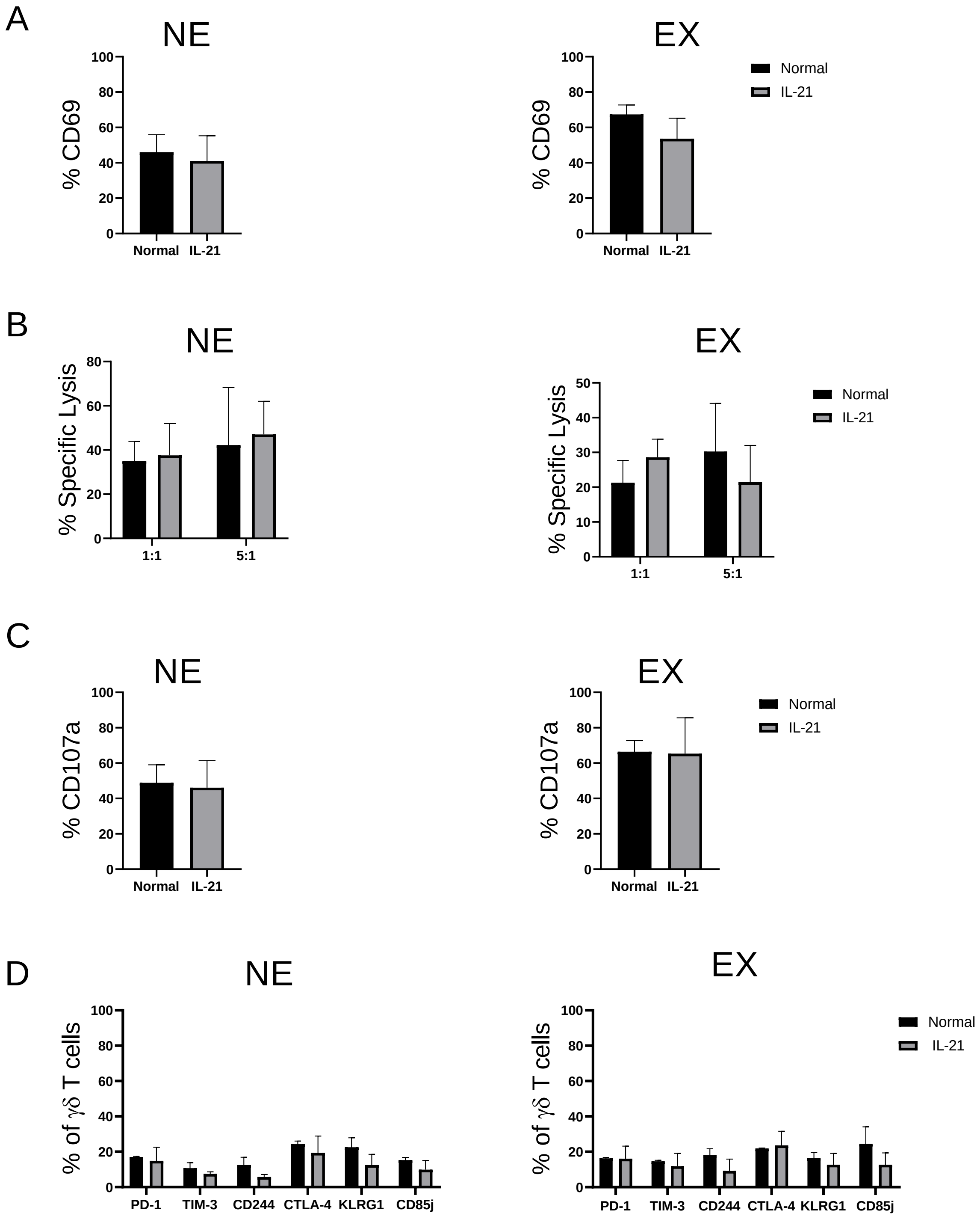

Supplemental Figure 5.

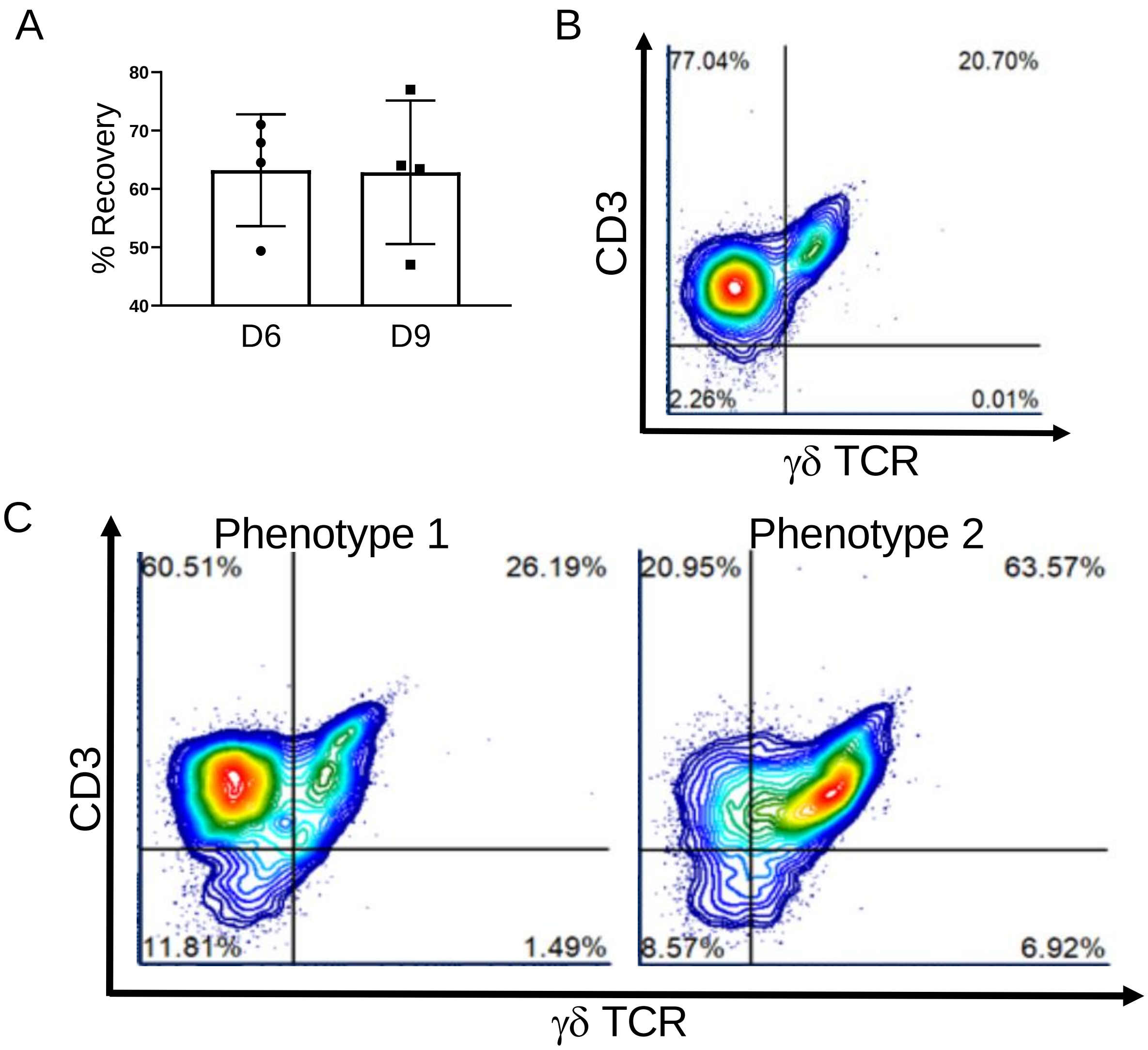

Supplemental Figure 6.

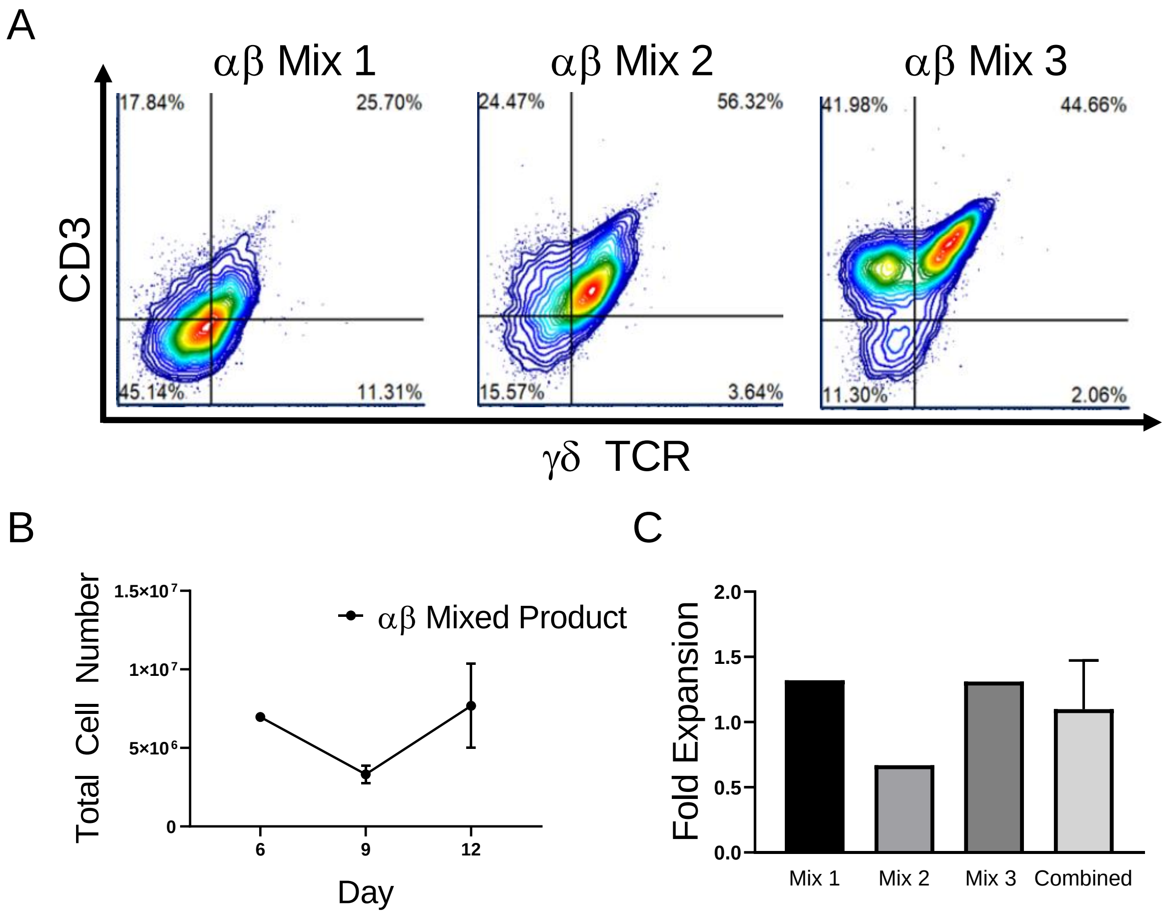

Supplemental Figure 7.

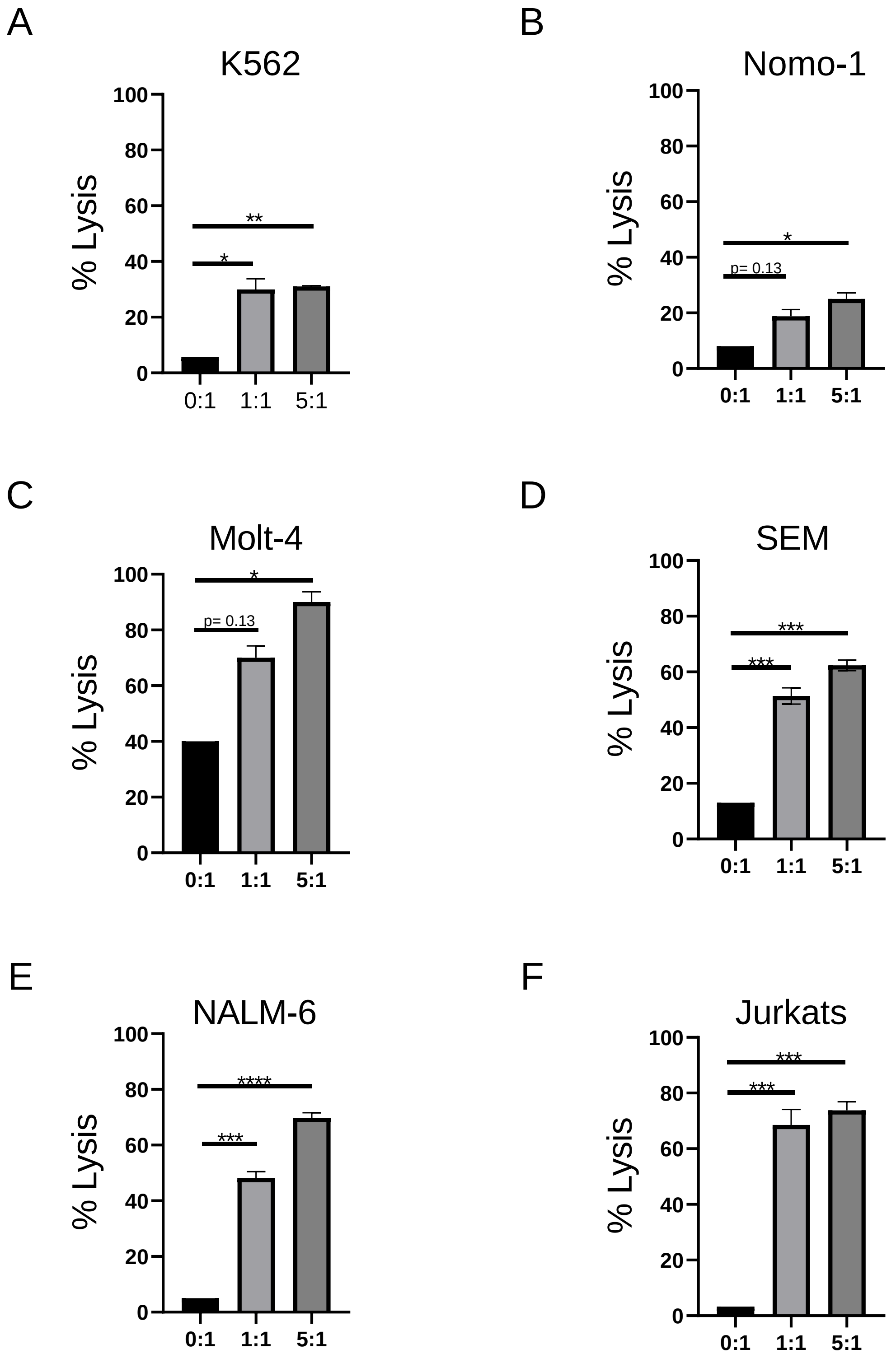

Supplement: Supplementary file 1 [file Data_Sheet_1.pdf]
